# Supplementary material for: Click Beetle Mitogenomics with the Definition of a New Subfamily Hapatesinae from Australasia (Coleoptera: Elateridae)
Source: Insects. 2020 Dec 29;12(1):17. doi: 10.3390/insects12010017 (PMC7859858; doi:10.3390/insects12010017)
Supplement: Supplementary file 1 [file insects-12-00017-s001.pdf]

Electronic supplementary information

Click beetle mitogenomics with the definition of a new subfamily Hapatesinae from Australasia (Coleoptera: Elateridae)

Dominik Kusy, Michal Motyka & Ladislav Bocak

Supplementary Tables

Table S1. The list of earlier reported taxa included in the present mitogenomic datasets with the number of genes included in the analysis.

Table S2. Location of features in the newly sequenced mitogenomes.

Table S3. Detailed overview of analysed datasets with partition schemes and results of the ModelFinder analysis.

Supplementary Illustrations

Figure S1. Newly sequenced circularized mitogenomes. The grey circles inside each mitogenomes represent GC content.

Figure S2. Gene order in newly sequenced linear mitogenomes.

Figure S3. AliStat heat maps of pairwise completeness scores (Ca) in all produced datasets: (A) NUC123: 15 mitochondrial genes, (B) PCN12: 13 mitochondrial PCGs, (C) AA: amino acids of 13 mitochondrial PCGs, (D) MTallAS: dataset A analysed using AliScore.

Figure S4. Heat maps calculated with SymTest showing p-values for the pairwise Bowker's tests in in all produced datasets: (A) NUC123: 15 mitochondrial genes, (B) PCN12: 13 mitochondrial PCGs, (C) AA: amino acids of 13 mitochondrial PCGs, (D) MTallAS: dataset A analysed using AliScore.

Figure S5. Rectangular heat maps of heterogeneous sequence divergence within sequences in analyzed datasets: (A) NUC123: 15 mitochondrial genes, (B) PCN12: 13 mitochondrial PCGs, (C) AA: amino acids of 13 mitochondrial PCGs, (D) MTallAS: dataset A analysed using AliScore, (E) 13 PCGs third codon position. The mean similarity score between sequences is represented by a coloured square, based on AliGROOVE scores ranging from −1, indicating great difference in rates from the remainder of the data set, i.e. heterogeneity (red), to +1, indicating rates match all other comparisons (blue).

Figure S6. Maximum likelihood trees from IQ-TREE analysis of datasets: A) NUC123: 15 mitochondrial genes partitioned by gene and PCGs further partitioned by codon position; B) PCN12: 13 mitochondrial PCGs partitioned by gene and by first and second codon positions with third codon position removed; C) AA: amino acids of 13 mitochondrial PCGs partitioned by gene; D) MTallAS: dataset A analysed using AliScore. The depicted branch support values represent SH-aLRT, abayes test, and ultrafast bootstrap.

Figure S7. Bayesian trees inferred from unpartitioned datasets: (A) NUC123: 15 mitochondrial genes, (B) PCN12: 13 mitochondrial PCGs and (C) AA: amino acids of 13 mitochondrial PCGs in PhyloBayes under the site-heterogeneous mixture CAT+ GTR model. The values at nodes are Bayesian posterior probabilities.

Table S1. The list of earlier reported taxa included in the present mitogenomic datasets with the number of genes included in the analysis.

	NCBI Voucher	Species	Subfamily	Family	# of genes
**Outgroup**	MH065615	*Sinopyrophorus schimmeli*	Sinopyrophorinae	Sinopyrophoridae	15
**Ingroup**	KT876879	*Agriotes obscurus*	Elaterinae	Elateridae	14
	JX412737	*Agriotes ustulatus*	Elaterinae	Elateridae	12
	KX087232	*Adrastus rachifer*	Elaterinae	Elateridae	15
	MN306531	*Ludioschema vittiger*	Elaterinae	Elateridae	15
	MH789726	Elateridae sp.	Elaterinae	Elateridae	15
	KT876904	*Melanotus villosus*	Elaterinae	Elateridae	15
	KJ922150	*Pyrearinus termitilluminans*	Agrypninae	Elateridae	15
	EF398270	*Pyrophorus divergens*	Agrypninae	Elateridae	13
	MG242621	*Ignelater luminosus*	Agrypninae	Elateridae	15
	KJ922149	*Hapsodrilus ignifer*	Agrypninae	Elateridae	15
	MK524933	*Cryptalaus yamato*	Agrypninae	Elateridae	15
	MT118665	*Cryptalaus larvatus*	Agrypninae	Elateridae	15
	MN370897	*Agrypnus* sp.	Agrypninae	Elateridae	15
	HQ232815	*Drilus flavescens*	Agrypninae	Elateridae	13
	KX087237	*Anostirus castaneus*	Dendrometrinae	Elateridae	15
	KX087306	*Limonius minutus*	Dendrometrinae	Elateridae	15
	KT852377	*Limonius californicus*	Dendrometrinae	Elateridae	15
	KT876881	*Athous haemorrhoidalis*	Dendrometrinae	Elateridae	15
	MK692585	*Cardiophorus signatus*	Cardiphorinae	Elateridae	15
	KX087283	*Dicronychus cinereus*	Cardiphorinae	Elateridae	15
	JX412848	*Dicronychus* sp.	Cardiphorinae	Elateridae	12
	KJ938491	*Teslasena femoralis*	Cardiphorinae	Elateridae	15

Table S2. Location of features in the newly sequenced mitogenomes.

Taxon	Name	Type	Minimum	Maximum	Length	Direction
D19007 Eudicronychus rufus	rrnL	rRNA	12,547	13,832	1,286	reverse
D19007 Eudicronychus rufus	rrnS	rRNA	13,903	14,659	757	reverse
D19007 Eudicronychus rufus	nad5	gene	6,254	7,990	1,737	reverse
D19007 Eudicronychus rufus	cox1	gene	1,399	2,961	1,563	forward
D19007 Eudicronychus rufus	nad4	gene	8,056	9,387	1,332	reverse
D19007 Eudicronychus rufus	cob	gene	10,310	11,443	1,134	forward
D19007 Eudicronychus rufus	nad2	gene	201	1,229	1,029	forward
D19007 Eudicronychus rufus	nad1	gene	11,531	12,481	951	reverse
D19007 Eudicronychus rufus	cox3	gene	4,672	5,520	849	forward
D19007 Eudicronychus rufus	cox2	gene	3,033	3,746	714	forward
D19007 Eudicronychus rufus	atp6	gene	3,998	4,672	675	forward
D19007 Eudicronychus rufus	nad6	gene	9,804	10,310	507	forward
D19007 Eudicronychus rufus	nad3	gene	5,521	5,877	357	forward
D19007 Eudicronychus rufus	nad4l	gene	9,381	9,671	291	reverse
D19007 Eudicronychus rufus	atp8	gene	3,849	4,004	156	forward
D19007 Eudicronychus rufus	trnW(tca)	tRNA	1,235	1,306	72	forward
D19007 Eudicronychus rufus	trnS2(tga)	tRNA	11,442	11,513	72	forward
D19007 Eudicronychus rufus	trnK(ctt)	tRNA	3,712	3,782	71	forward
D19007 Eudicronychus rufus	trnV(tac)	tRNA	13,833	13,902	70	reverse
D19007 Eudicronychus rufus	trnQ(ttg)	tRNA	65	133	69	reverse
D19007 Eudicronychus rufus	trnM(cat)	tRNA	133	200	68	forward
D19007 Eudicronychus rufus	trnA(tgc)	tRNA	5,876	5,943	68	forward
D19007 Eudicronychus rufus	trnI(gat)	tRNA	1	67	67	forward
D19007 Eudicronychus rufus	trnY(gta)	tRNA	1,361	1,427	67	reverse
D19007 Eudicronychus rufus	trnD(gtc)	tRNA	3,782	3,848	67	forward
D19007 Eudicronychus rufus	trnS1(tct)	tRNA	6,074	6,140	67	forward
D19007 Eudicronychus rufus	trnE(ttc)	tRNA	6,142	6,208	67	forward
D19007 Eudicronychus rufus	trnF(gaa)	tRNA	6,207	6,273	67	reverse
D19007 Eudicronychus rufus	trnR(tcg)	tRNA	5,943	6,008	66	forward
D19007 Eudicronychus rufus	trnN(gtt)	tRNA	6,008	6,073	66	forward
D19007 Eudicronychus rufus	trnH(gtg)	tRNA	7,991	8,056	66	reverse
D19007 Eudicronychus rufus	trnP(tgg)	tRNA	9,738	9,802	65	reverse
D19007 Eudicronychus rufus	trnL2(taa)	tRNA	2,965	3,028	64	forward
D19007 Eudicronychus rufus	trnT(tgt)	tRNA	9,674	9,737	64	forward
D19007 Eudicronychus rufus	trnL1(tag)	tRNA	12,483	12,545	63	reverse
D19007 Eudicronychus rufus	trnC(gca)	tRNA	1,299	1,360	62	reverse
G19011 Diplophoenicus sp.	rrnL	rRNA	12,521	13,802	1,282	reverse
G19011 Diplophoenicus sp.	rrnS	rRNA	13,875	14,630	756	reverse
G19011 Diplophoenicus sp.	nad5	gene	6,263	7,978	1,716	reverse
G19011 Diplophoenicus sp.	cox1	gene	1,417	3,000	1,584	forward
G19011 Diplophoenicus sp.	nad4	gene	7,978	9,324	1,347	reverse
G19011 Diplophoenicus sp.	cob	gene	10,292	11,425	1,134	forward
G19011 Diplophoenicus sp.	nad2	gene	196	1,224	1,029	forward
G19011 Diplophoenicus sp.	nad1	gene	11,507	12,457	951	reverse
G19011 Diplophoenicus sp.	cox3	gene	4,668	5,516	849	forward
G19011 Diplophoenicus sp.	cox2	gene	3,032	3,745	714	forward
G19011 Diplophoenicus sp.	atp6	gene	3,994	4,668	675	forward
G19011 Diplophoenicus sp.	nad6	gene	9,777	10,292	516	forward
G19011 Diplophoenicus sp.	nad3	gene	5,517	5,873	357	forward
G19011 Diplophoenicus sp.	nad4l	gene	9,366	9,656	291	reverse
G19011 Diplophoenicus sp.	atp8	gene	3,845	4,000	156	forward
G19011 Diplophoenicus sp.	trnV(tac)	tRNA	13,803	13,874	72	reverse
G19011 Diplophoenicus sp.	trnK(ctt)	tRNA	3,711	3,781	71	forward
G19011 Diplophoenicus sp.	trnQ(ttg)	tRNA	61	129	69	reverse
G19011 Diplophoenicus sp.	trnS1(tct)	tRNA	6,064	6,131	68	forward
G19011 Diplophoenicus sp.	trnM(cat)	tRNA	129	195	67	forward
G19011 Diplophoenicus sp.	trnW(tca)	tRNA	1,227	1,293	67	forward
G19011 Diplophoenicus sp.	trnF(gaa)	tRNA	6,197	6,263	67	reverse
G19011 Diplophoenicus sp.	trnA(tgc)	tRNA	5,872	5,937	66	forward
G19011 Diplophoenicus sp.	trnE(ttc)	tRNA	6,133	6,198	66	forward
G19011 Diplophoenicus sp.	trnS2(tga)	tRNA	11,424	11,489	66	forward
G19011 Diplophoenicus sp.	trnN(gtt)	tRNA	5,999	6,063	65	forward
G19011 Diplophoenicus sp.	trnY(gta)	tRNA	1,349	1,412	64	reverse
G19011 Diplophoenicus sp.	trnD(gtc)	tRNA	3,781	3,844	64	forward
G19011 Diplophoenicus sp.	trnP(tgg)	tRNA	9,721	9,784	64	reverse
G19011 Diplophoenicus sp.	trnI(gat)	tRNA	1	63	63	forward
G19011 Diplophoenicus sp.	trnC(gca)	tRNA	1,286	1,348	63	reverse
G19011 Diplophoenicus sp.	trnL2(taa)	tRNA	2,969	3,031	63	forward
G19011 Diplophoenicus sp.	trnR(tcg)	tRNA	5,937	5,999	63	forward
G19011 Diplophoenicus sp.	trnT(tgt)	tRNA	9,658	9,720	63	forward
G19011 Diplophoenicus sp.	trnL1(tag)	tRNA	12,459	12,520	62	reverse
G18004 Drilus mauritanicus	rrnL	rRNA	12,422	13,708	1,287	reverse
G18004 Drilus mauritanicus	rrnS	rRNA	13,761	14,507	747	reverse
G18004 Drilus mauritanicus	nad5	gene	6,207	7,927	1,721	reverse
G18004 Drilus mauritanicus	cox1	gene	1,376	2,937	1,562	forward
G18004 Drilus mauritanicus	nad4	gene	7,924	9,306	1,383	reverse
G18004 Drilus mauritanicus	cob	gene	10,206	11,336	1,131	forward
G18004 Drilus mauritanicus	nad2	gene	197	1,216	1,020	forward
G18004 Drilus mauritanicus	nad1	gene	11,415	12,365	951	reverse
G18004 Drilus mauritanicus	cox3	gene	4,629	5,474	846	forward
G18004 Drilus mauritanicus	cox2	gene	3,001	3,685	685	forward
G18004 Drilus mauritanicus	atp6	gene	3,955	4,626	672	forward
G18004 Drilus mauritanicus	nad6	gene	9,706	10,206	501	forward
G18004 Drilus mauritanicus	nad3	gene	5,475	5,831	357	forward
G18004 Drilus mauritanicus	nad4l	gene	9,300	9,587	288	reverse
G18004 Drilus mauritanicus	atp8	gene	3,806	3,961	156	forward
G18004 Drilus mauritanicus	trnK(ctt)	tRNA	3,674	3,743	70	forward
G18004 Drilus mauritanicus	trnQ(ttg)	tRNA	63	131	69	reverse
G18004 Drilus mauritanicus	trnV(tac)	tRNA	13,693	13,761	69	reverse
G18004 Drilus mauritanicus	trnW(tca)	tRNA	1,222	1,289	68	forward
G18004 Drilus mauritanicus	trnM(cat)	tRNA	131	196	66	forward
G18004 Drilus mauritanicus	trnE(ttc)	tRNA	6,082	6,147	66	forward
G18004 Drilus mauritanicus	trnI(gat)	tRNA	1	65	65	forward
G18004 Drilus mauritanicus	trnS1(tct)	tRNA	6,017	6,081	65	forward
G18004 Drilus mauritanicus	trnL2(taa)	tRNA	2,938	3,001	64	forward
G18004 Drilus mauritanicus	trnN(gtt)	tRNA	5,953	6,016	64	forward
G18004 Drilus mauritanicus	trnD(gtc)	tRNA	3,743	3,805	63	forward
G18004 Drilus mauritanicus	trnA(tgc)	tRNA	5,830	5,892	63	forward
G18004 Drilus mauritanicus	trnT(tgt)	tRNA	9,589	9,651	63	forward
G18004 Drilus mauritanicus	trnY(gta)	tRNA	1,343	1,404	62	reverse
G18004 Drilus mauritanicus	trnF(gaa)	tRNA	6,146	6,207	62	reverse
G18004 Drilus mauritanicus	trnP(tgg)	tRNA	9,652	9,713	62	reverse
G18004 Drilus mauritanicus	trnS2(tga)	tRNA	11,335	11,396	62	forward
G18004 Drilus mauritanicus	trnC(gca)	tRNA	1,282	1,342	61	reverse
G18004 Drilus mauritanicus	trnR(tcg)	tRNA	5,892	5,952	61	forward
G18004 Drilus mauritanicus	trnL1(tag)	tRNA	12,367	12,427	61	reverse
G20004 Eudicronychus sp.	rrnL	rRNA	12,548	13,832	1,285	reverse
G20004 Eudicronychus sp.	rrnS	rRNA	13,903	14,659	757	reverse
G20004 Eudicronychus sp.	nad5	gene	6,256	7,995	1,740	reverse
G20004 Eudicronychus sp.	cox1	gene	1,402	2,964	1,563	forward
G20004 Eudicronychus sp.	nad4	gene	8,058	9,389	1,332	reverse
G20004 Eudicronychus sp.	cob	gene	10,312	11,445	1,134	forward
G20004 Eudicronychus sp.	nad2	gene	202	1,230	1,029	forward
G20004 Eudicronychus sp.	nad1	gene	11,532	12,482	951	reverse
G20004 Eudicronychus sp.	cox3	gene	4,675	5,523	849	forward
G20004 Eudicronychus sp.	cox2	gene	3,036	3,749	714	forward
G20004 Eudicronychus sp.	atp6	gene	4,001	4,675	675	forward
G20004 Eudicronychus sp.	nad6	gene	9,806	10,312	507	forward
G20004 Eudicronychus sp.	nad3	gene	5,524	5,880	357	forward
G20004 Eudicronychus sp.	nad4l	gene	9,383	9,673	291	reverse
G20004 Eudicronychus sp.	atp8	gene	3,852	4,007	156	forward
G20004 Eudicronychus sp.	trnW(tca)	tRNA	1,236	1,308	73	forward
G20004 Eudicronychus sp.	trnK(ctt)	tRNA	3,715	3,785	71	forward
G20004 Eudicronychus sp.	trnS2(tga)	tRNA	11,444	11,514	71	forward
G20004 Eudicronychus sp.	trnV(tac)	tRNA	13,833	13,902	70	reverse
G20004 Eudicronychus sp.	trnQ(ttg)	tRNA	65	133	69	reverse
G20004 Eudicronychus sp.	trnM(cat)	tRNA	134	201	68	forward
G20004 Eudicronychus sp.	trnA(tgc)	tRNA	5,879	5,946	68	forward
G20004 Eudicronychus sp.	trnI(gat)	tRNA	1	67	67	forward
G20004 Eudicronychus sp.	trnY(gta)	tRNA	1,364	1,430	67	reverse
G20004 Eudicronychus sp.	trnD(gtc)	tRNA	3,785	3,851	67	forward
G20004 Eudicronychus sp.	trnS1(tct)	tRNA	6,077	6,143	67	forward
G20004 Eudicronychus sp.	trnE(ttc)	tRNA	6,145	6,211	67	forward
G20004 Eudicronychus sp.	trnR(tcg)	tRNA	5,946	6,011	66	forward
G20004 Eudicronychus sp.	trnN(gtt)	tRNA	6,011	6,076	66	forward
G20004 Eudicronychus sp.	trnF(gaa)	tRNA	6,210	6,275	66	reverse
G20004 Eudicronychus sp.	trnH(gtg)	tRNA	7,993	8,058	66	reverse
G20004 Eudicronychus sp.	trnP(tgg)	tRNA	9,740	9,804	65	reverse
G20004 Eudicronychus sp.	trnL2(taa)	tRNA	2,968	3,031	64	forward
G20004 Eudicronychus sp.	trnT(tgt)	tRNA	9,676	9,739	64	forward
G20004 Eudicronychus sp.	trnC(gca)	tRNA	1,301	1,363	63	reverse
G20004 Eudicronychus sp.	trnL1(tag)	tRNA	12,484	12,546	63	reverse

Taxon	Name	Type	Minimum	Maximum	Length	Direction
G20007 Hapatesus tropicus	rrnL	rRNA	12,504	13,828	1,325	reverse
G20007 Hapatesus tropicus	rrnS	rRNA	13,884	14,647	764	reverse
G20007 Hapatesus tropicus	nad5	gene	6,256	7,974	1,719	reverse
G20007 Hapatesus tropicus	cox1	gene	1,399	2,961	1,563	forward
G20007 Hapatesus tropicus	nad4	gene	8,042	9,373	1,332	reverse
G20007 Hapatesus tropicus	cob	gene	10,294	11,427	1,134	forward
G20007 Hapatesus tropicus	nad2	gene	199	1,224	1,026	forward
G20007 Hapatesus tropicus	nad1	gene	11,512	12,462	951	reverse
G20007 Hapatesus tropicus	cox3	gene	4,666	5,453	788	forward
G20007 Hapatesus tropicus	cox2	gene	3,029	3,742	714	forward
G20007 Hapatesus tropicus	atp6	gene	3,992	4,666	675	forward
G20007 Hapatesus tropicus	nad6	gene	9,788	10,294	507	forward
G20007 Hapatesus tropicus	nad3	gene	5,517	5,870	354	forward
G20007 Hapatesus tropicus	nad4l	gene	9,367	9,657	291	reverse
G20007 Hapatesus tropicus	atp8	gene	3,843	3,998	156	forward
G20007 Hapatesus tropicus	trnK(ctt)	tRNA	3,708	3,778	71	forward
G20007 Hapatesus tropicus	trnV(tac)	tRNA	13,813	13,883	71	reverse
G20007 Hapatesus tropicus	trnQ(ttg)	tRNA	64	132	69	reverse
G20007 Hapatesus tropicus	trnC(gca)	tRNA	1,291	1,358	68	reverse
G20007 Hapatesus tropicus	trnM(cat)	tRNA	132	198	67	forward
G20007 Hapatesus tropicus	trnS1(tct)	tRNA	6,063	6,129	67	forward
G20007 Hapatesus tropicus	trnI(gat)	tRNA	1	66	66	forward
G20007 Hapatesus tropicus	trnN(gtt)	tRNA	5,997	6,062	66	forward
G20007 Hapatesus tropicus	trnH(gtg)	tRNA	7,975	8,040	66	reverse
G20007 Hapatesus tropicus	trnS2(tga)	tRNA	11,426	11,491	66	forward
G20007 Hapatesus tropicus	trnY(gta)	tRNA	1,363	1,427	65	reverse
G20007 Hapatesus tropicus	trnD(gtc)	tRNA	3,778	3,842	65	forward
G20007 Hapatesus tropicus	trnA(tgc)	tRNA	5,869	5,933	65	forward
G20007 Hapatesus tropicus	trnR(tcg)	tRNA	5,932	5,996	65	forward
G20007 Hapatesus tropicus	trnE(ttc)	tRNA	6,131	6,195	65	forward
G20007 Hapatesus tropicus	trnP(tgg)	tRNA	9,722	9,786	65	reverse
G20007 Hapatesus tropicus	trnL2(taa)	tRNA	2,964	3,027	64	forward
G20007 Hapatesus tropicus	trnG(tcc)	tRNA	5,453	5,516	64	forward
G20007 Hapatesus tropicus	trnT(tgt)	tRNA	9,659	9,722	64	forward
G20007 Hapatesus tropicus	trnF(gaa)	tRNA	6,194	6,256	63	reverse
G20007 Hapatesus tropicus	trnL1(tag)	tRNA	12,464	12,526	63	reverse
G20007 Hapatesus tropicus	trnW(tca)	tRNA	1,230	1,283	54	forward
G19006 Parablax sp.	rrnL	rRNA	12,517	13,844	1,328	reverse
G19006 Parablax sp.	rrnS	rRNA	13,893	14,653	761	reverse
G19006 Parablax sp.	nad5	gene	6,256	7,929	1,674	reverse
G19006 Parablax sp.	cox1	gene	1,439	2,980	1,542	forward
G19006 Parablax sp.	nad4	gene	8,052	9,387	1,336	reverse
G19006 Parablax sp.	cob	gene	10,310	11,443	1,134	forward
G19006 Parablax sp.	nad2	gene	222	1,250	1,029	forward
G19006 Parablax sp.	nad1	gene	11,525	12,475	951	reverse
G19006 Parablax sp.	cox3	gene	4,683	5,470	788	forward
G19006 Parablax sp.	cox2	gene	3,046	3,759	714	forward
G19006 Parablax sp.	atp6	gene	4,009	4,683	675	forward
G19006 Parablax sp.	nad6	gene	9,804	10,310	507	forward
G19006 Parablax sp.	nad3	gene	5,534	5,887	354	forward
G19006 Parablax sp.	nad4l	gene	9,381	9,671	291	reverse
G19006 Parablax sp.	atp8	gene	3,860	4,015	156	forward
G19006 Parablax sp.	trnW(tca)	tRNA	1,257	1,327	71	forward
G19006 Parablax sp.	trnK(ctt)	tRNA	3,725	3,795	71	forward
G19006 Parablax sp.	trnV(tac)	tRNA	13,823	13,892	70	reverse
G19006 Parablax sp.	trnQ(ttg)	tRNA	64	132	69	reverse
G19006 Parablax sp.	trnR(tcg)	tRNA	5,950	6,016	67	forward
G19006 Parablax sp.	trnS1(tct)	tRNA	6,086	6,152	67	forward
G19006 Parablax sp.	trnI(gat)	tRNA	1	66	66	forward
G19006 Parablax sp.	trnM(cat)	tRNA	156	221	66	forward
G19006 Parablax sp.	trnN(gtt)	tRNA	6,020	6,085	66	forward
G19006 Parablax sp.	trnE(ttc)	tRNA	6,155	6,220	66	forward
G19006 Parablax sp.	trnP(tgg)	tRNA	9,737	9,802	66	reverse
G19006 Parablax sp.	trnS2(tga)	tRNA	11,442	11,507	66	forward
G19006 Parablax sp.	trnY(gta)	tRNA	1,382	1,446	65	reverse
G19006 Parablax sp.	trnD(gtc)	tRNA	3,795	3,859	65	forward
G19006 Parablax sp.	trnA(tgc)	tRNA	5,886	5,950	65	forward
G19006 Parablax sp.	trnH(gtg)	tRNA	7,999	8,063	65	reverse
G19006 Parablax sp.	trnL2(taa)	tRNA	2,982	3,045	64	forward
G19006 Parablax sp.	trnG(tcc)	tRNA	5,470	5,533	64	forward
G19006 Parablax sp.	trnF(gaa)	tRNA	6,219	6,281	63	reverse
G19006 Parablax sp.	trnL1(tag)	tRNA	12,477	12,539	63	reverse
G19006 Parablax sp.	trnC(gca)	tRNA	1,320	1,381	62	reverse
G19006 Parablax sp.	trnT(tgt)	tRNA	9,675	9,736	62	forward
A01544 Plastocerus angulosus	rrnL	rRNA	12,532	13,840	1,309	reverse
A01544 Plastocerus angulosus	rrnS	rRNA	13,892	14,659	768	reverse
A01544 Plastocerus angulosus	nad5	gene	6,242	7,987	1,746	reverse
A01544 Plastocerus angulosus	cox1	gene	1,418	2,957	1,540	forward
A01544 Plastocerus angulosus	nad4	gene	8,047	9,376	1,330	reverse
A01544 Plastocerus angulosus	cob	gene	10,298	11,431	1,134	forward
A01544 Plastocerus angulosus	nad2	gene	200	1,228	1,029	forward
A01544 Plastocerus angulosus	nad1	gene	11,515	12,465	951	reverse
A01544 Plastocerus angulosus	cox3	gene	4,663	5,448	786	forward
A01544 Plastocerus angulosus	cox2	gene	3,025	3,703	679	forward
A01544 Plastocerus angulosus	atp6	gene	3,989	4,663	675	forward
A01544 Plastocerus angulosus	nad6	gene	9,783	10,298	516	forward
A01544 Plastocerus angulosus	nad3	gene	5,515	5,868	354	forward
A01544 Plastocerus angulosus	nad4l	gene	9,370	9,660	291	reverse
A01544 Plastocerus angulosus	atp8	gene	3,840	3,995	156	forward
A01544 Plastocerus angulosus	trnW(tca)	tRNA	1,235	1,307	73	forward
A01544 Plastocerus angulosus	trnK(ctt)	tRNA	3,704	3,774	71	forward
A01544 Plastocerus angulosus	trnR(tcg)	tRNA	5,932	6,002	71	forward
A01544 Plastocerus angulosus	trnV(tac)	tRNA	13,822	13,891	70	reverse
A01544 Plastocerus angulosus	trnQ(ttg)	tRNA	65	133	69	reverse
A01544 Plastocerus angulosus	trnX(tct)	tRNA	6,068	6,135	68	forward
A01544 Plastocerus angulosus	trnS2(tga)	tRNA	11,430	11,497	68	forward
A01544 Plastocerus angulosus	trnI(gat)	tRNA	1	67	67	forward
A01544 Plastocerus angulosus	trnM(cat)	tRNA	133	199	67	forward
A01544 Plastocerus angulosus	trnD(gtc)	tRNA	3,774	3,839	66	forward
A01544 Plastocerus angulosus	trnA(tgc)	tRNA	5,867	5,932	66	forward
A01544 Plastocerus angulosus	trnN(gtt)	tRNA	6,002	6,067	66	forward
A01544 Plastocerus angulosus	trnE(ttc)	tRNA	6,139	6,204	66	forward
A01544 Plastocerus angulosus	trnY(gta)	tRNA	1,361	1,425	65	reverse
A01544 Plastocerus angulosus	trnG(tcc)	tRNA	5,450	5,514	65	forward
A01544 Plastocerus angulosus	trnF(gaa)	tRNA	6,203	6,267	65	reverse
A01544 Plastocerus angulosus	trnH(---)	tRNA	7,985	8,049	65	reverse
A01544 Plastocerus angulosus	trnL1(tag)	tRNA	12,467	12,531	65	reverse
A01544 Plastocerus angulosus	trnT(tgt)	tRNA	9,663	9,726	64	forward
A01544 Plastocerus angulosus	trnP(tgg)	tRNA	9,727	9,790	64	reverse
A01544 Plastocerus angulosus	trnL2(taa)	tRNA	2,958	3,020	63	forward
A01544 Plastocerus angulosus	trnC(gca)	tRNA	1,300	1,360	61	reverse
G20012 Tibionema abdominalis	rrnL	rRNA	12,518	13,798	1,281	reverse
G20012 Tibionema abdominalis	rrnS	rRNA	13,869	14,624	756	reverse
G20012 Tibionema abdominalis	nad5	gene	6,231	7,970	1,740	reverse
G20012 Tibionema abdominalis	cox1	gene	1,413	2,954	1,542	forward
G20012 Tibionema abdominalis	nad4	gene	7,967	9,358	1,392	reverse
G20012 Tibionema abdominalis	cob	gene	10,287	11,420	1,134	forward
G20012 Tibionema abdominalis	nad2	gene	201	1,226	1,026	forward
G20012 Tibionema abdominalis	nad1	gene	11,504	12,454	951	reverse
G20012 Tibionema abdominalis	cox3	gene	4,666	5,453	788	forward
G20012 Tibionema abdominalis	cox2	gene	3,021	3,737	717	forward
G20012 Tibionema abdominalis	atp6	gene	3,992	4,666	675	forward
G20012 Tibionema abdominalis	nad6	gene	9,778	10,287	510	forward
G20012 Tibionema abdominalis	nad3	gene	5,516	5,869	354	forward
G20012 Tibionema abdominalis	nad4l	gene	9,355	9,645	291	reverse
G20012 Tibionema abdominalis	atp8	gene	3,843	3,998	156	forward
G20012 Tibionema abdominalis	trnK(ctt)	tRNA	3,703	3,773	71	forward
G20012 Tibionema abdominalis	trnS1(tct)	tRNA	6,062	6,131	70	forward
G20012 Tibionema abdominalis	trnV(tac)	tRNA	13,799	13,868	70	reverse
G20012 Tibionema abdominalis	trnQ(ttg)	tRNA	65	133	69	reverse
G20012 Tibionema abdominalis	trnW(tca)	tRNA	1,234	1,302	69	forward
G20012 Tibionema abdominalis	trnS2(tga)	tRNA	11,419	11,486	68	forward
G20012 Tibionema abdominalis	trnI(gat)	tRNA	1	67	67	forward
G20012 Tibionema abdominalis	trnM(cat)	tRNA	135	200	66	forward
G20012 Tibionema abdominalis	trnD(gtc)	tRNA	3,777	3,842	66	forward
G20012 Tibionema abdominalis	trnP(tgg)	tRNA	9,711	9,776	66	reverse
G20012 Tibionema abdominalis	trnA(tgc)	tRNA	5,868	5,932	65	forward
G20012 Tibionema abdominalis	trnN(gtt)	tRNA	5,997	6,061	65	forward
G20012 Tibionema abdominalis	trnY(gta)	tRNA	1,357	1,420	64	reverse
G20012 Tibionema abdominalis	trnF(gaa)	tRNA	6,193	6,256	64	reverse
G20012 Tibionema abdominalis	trnT(tgt)	tRNA	9,647	9,710	64	forward
G20012 Tibionema abdominalis	trnC(gca)	tRNA	1,295	1,357	63	reverse
G20012 Tibionema abdominalis	trnL2(taa)	tRNA	2,959	3,021	63	forward
G20012 Tibionema abdominalis	trnG(tcc)	tRNA	5,453	5,515	63	forward
G20012 Tibionema abdominalis	trnE(ttc)	tRNA	6,132	6,194	63	forward
G20012 Tibionema abdominalis	trnR(tcg)	tRNA	5,932	5,993	62	forward
G20012 Tibionema abdominalis	trnL1(tag)	tRNA	12,456	12,517	62	reverse

Table S3. Detailed overview of analysed datasets with partition schemes and results of the ModelFinder analysis.

Dataset A) 15 mitochondrial genes partitioned by gene and PCGs further partitioned by codon position.
Input data: 31 taxa with 41 partitions and 13341 total sites (2.29296% missing data)

Gene	Seq	Site	Unique	Infor	Invar	Const	ModelFinder
12S	28	820	486	372	347	347	TPM3+F+I+G4
16S	28	1331	825	669	511	510	TPM3+F+R4
atp6	30	224	110	93	116	116	TIM3+F+I+G4
atp6b	30	224	57	36	171	171	TVM+F+G4
atp6c	30	224	217	204	9	9	TN+F+G4
atp8	29	51	39	32	15	15	K3Pu+F+G4
atp8b	29	51	34	24	21	21	TPM3+F+G4
atp8c	29	51	48	46	4	4	TIM3+F+G4
cox1	31	520	173	118	374	374	SYM+I+G4
cox1b	31	520	73	29	474	474	TVM+F+R2
cox1c	31	520	513	485	15	15	TPM3+F+R4
cox2	31	226	111	87	119	119	GTR+F+I+G4
cox2b	31	226	68	44	164	164	TPM3+F+I+G4
cox2c	31	226	224	220	4	4	TPM3+F+I+G4
cox3	31	262	113	89	150	150	TIM2+F+G4
cox3b	31	262	60	38	204	204	TVM+F+I+G4
cox3c	31	262	250	242	15	15	TPM3+F+I+G4
cytb	31	381	176	136	210	210	GTR+F+I+G4
cytbb	31	381	109	62	281	281	TIM3+F+I+G4
cytbc	31	381	375	349	10	10	HKY+F+I+G4
nad1	31	316	169	134	151	151	GTR+F+I+G4
nad1b	31	316	92	53	226	226	TVM+F+G4
nad1c	31	316	304	282	9	9	HKY+F+I+G4
nad2	29	342	237	208	113	113	GTR+F+I+G4
nad2b	29	342	149	113	188	188	GTR+F+I+G4
nad2c	29	342	339	325	5	5	HKY+F+R4
nad3	31	118	72	55	52	52	TIM3+F+I+G4
nad3b	31	118	53	34	71	71	TVM+F+G4
nad3c	31	118	116	113	4	4	HKY+F+G4
nad4	31	443	271	224	179	179	GTR+F+I+G4
nad4b	31	443	169	120	281	281	GTR+F+I+G4
nad4c	31	443	429	395	16	15	TPM2+F+R4
nad4l	31	100	65	51	40	40	TPM3+F+G4
nad4lb	31	100	49	26	60	60	TVM+F+G4
nad4lc	31	100	94	80	9	9	HKY+F+G4
nad5	31	575	317	268	256	256	GTR+F+I+G4
nad5b	31	575	206	150	375	375	GTR+F+I+G4
nad5c	31	575	545	506	25	25	HKY+F+G4
nad6	31	172	132	110	47	47	GTR+F+I+G4
nad6b	31	172	107	83	73	73	TVM+F+I+G4
nad6c	31	172	172	158	3	3	HKY+F+I+G4

Legend:
Unique: Number of unique site patterns
Infor: Number of parsimony-informative sites
Invar: Number of invariant sites
Const: Number of constant sites (can be subset of invariant sites)

Dataset B) 13 mitochondrial PCGs partitioned by gene and by first and second codon positions with third codon position removed.
Input data: 31 taxa with 26 partitions and 7460 total sites (0.873476% missing data)

Gene	Seq	Site	Unique	Infor	Invar	Const	ModelFinder
atp6	30	224	110	93	116	116	TIM3+F+I+G4
atp6b	30	224	57	36	171	171	TVM+F+G4
atp8	29	51	39	32	15	15	K3Pu+F+G4
atp8b	29	51	34	24	21	21	TPM3+F+G4
cox1	31	520	173	118	374	374	SYM+I+G4
cox1b	31	520	73	29	474	474	TVM+F+R2
cox2	31	226	111	87	119	119	TIM3+F+I+G4
cox2b	31	226	68	44	164	164	TPM3+F+I+G4
cox3	31	262	113	89	150	150	TIM2+F+G4
cox3b	31	262	60	38	204	204	TVM+F+I+G4
cytb	31	381	176	136	210	210	GTR+F+I+G4
cytbb	31	381	109	62	281	281	TIM3+F+I+G4
nad1	31	316	169	134	151	151	TIM2+F+I+G4
nad1b	31	316	92	53	226	226	TVM+F+G4
nad2	29	342	237	208	113	113	GTR+F+I+G4
nad2b	29	342	149	113	188	188	GTR+F+I+G4
nad3	31	118	72	55	52	52	TIM3+F+I+G4

Legend:
Unique: Number of unique site patterns
Infor: Number of parsimony-informative sites
Invar: Number of invariant sites
Const: Number of constant sites (can be subset of invariant sites)

nad3b	31	118	53	34	71	71	TVM+F+G4
nad4	31	443	271	224	179	179	TIM3+F+I+G4
nad4b	31	443	169	120	281	281	GTR+F+I+G4
nad4l	31	100	65	51	40	40	TPM3+F+G4
nad4lb	31	100	49	26	60	60	TVM+F+G4
nad5	31	575	317	268	256	256	GTR+F+I+G4
nad5b	31	575	206	150	375	375	GTR+F+I+G4
nad6	31	172	132	110	47	47	GTR+F+I+G4
nad6b	31	172	107	83	73	73	TVM+F+I+G4

Dataset C) Amino acids of 13 mitochondrial PCGs partitioned by gene.
Input data: 31 taxa with 13 partitions and 3730 total sites (0.873476% missing data)

Gene	Seq	Site	Unique	Infor	Invar	Const	ModelFinder
atp6	30	224	118	78	126	126	mtART+I+G4
atp8	29	51	46	34	14	14	mtMet+G4
cox1	31	520	180	78	409	409	mtZOA+I+G4
cox2	31	226	136	88	113	113	mtMet+R3
cox3	31	262	131	87	150	150	mtZOA+I+G4
cytb	31	381	211	131	197	197	mtZOA+G4
nad1	31	316	181	124	154	154	mtZOA+F+I+G4
nad2	29	342	263	212	101	101	mtZOA+F+R4
nad3	31	118	81	52	56	56	mtMet+G4
nad4	31	443	303	214	180	180	mtZOA+F+R4
nad4l	31	100	82	47	40	40	mtInv+G4
nad5	31	575	347	260	254	254	mtInv+F+R4
nad6	31	172	151	115	39	39	mtMet+F+G4

Legend:
Unique: Number of unique site patterns
Infor: Number of parsimony-informative sites
Invar: Number of invariant sites
Const: Number of constant sites (can be subset of invariant sites)

Dataset D) Dataset A analysed using AliScore and AliCut software to remove possibly ambiguously aligned regions.
Input data: 31 taxa with 15 partitions and 12613 total sites (2.24397% missing data)

Gene	Seq	Site	Unique	Infor	Invar	Const	ModelFinder
12S	28	795	461	349	346	346	TPM3+F+I+G4
cox3	31	768	397	351	369	369	GTR+F+I+G4
cytb	31	1126	620	530	501	501	GTR+F+I+G4
nad2	29	910	600	534	305	305	TPM3+F+R4
nad4l	31	287	182	145	109	109	K3Pu+F+G4
atp6	30	654	358	316	295	295	TIM+F+I+G4
nad4	31	1259	776	671	475	475	TIM+F+I+G4
nad5	31	1631	945	836	653	653	TIM+F+I+G4
nad1	31	927	526	448	386	386	TIM+F+I+G4
nad3	31	315	196	164	127	127	GTR+F+I+G4
cox2	31	653	364	326	287	287	GTR+F+I+G4
nad6	31	388	272	223	123	123	TN+F+I+G4
16S	28	1229	724	572	510	509	GTR+F+I+G4
cox1	31	1557	719	629	863	863	GTR+F+I+G4
atp8	29	114	78	63	40	40	HKY+F+I+G4

Legend:
Unique: Number of unique site patterns
Infor: Number of parsimony-informative sites
Invar: Number of invariant sites
Const: Number of constant sites (can be subset of invariant sites)

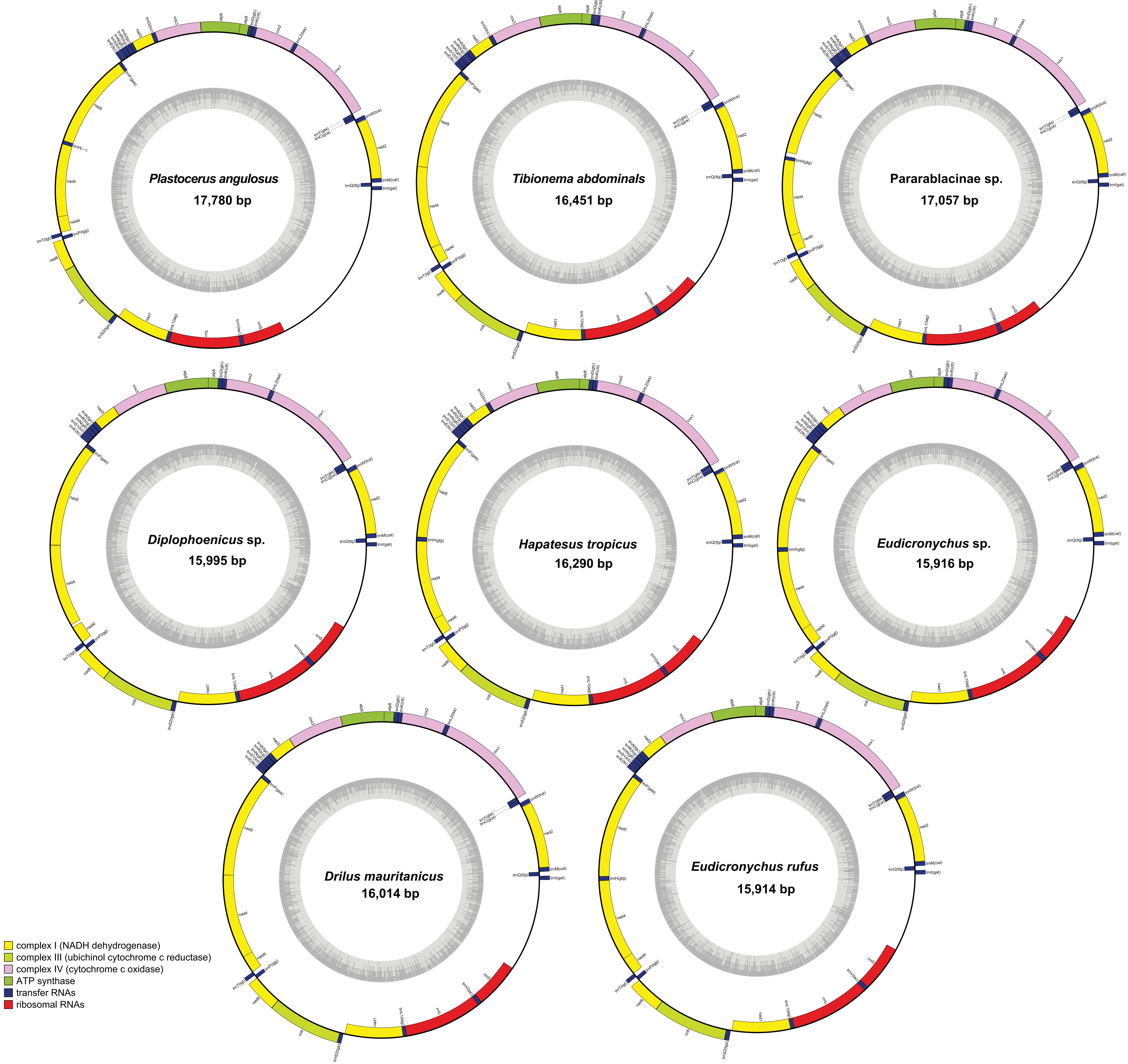

Figure S1. Newly sequenced circularized mitogenomes. The grey circles inside each mitogenomes represent GC content.

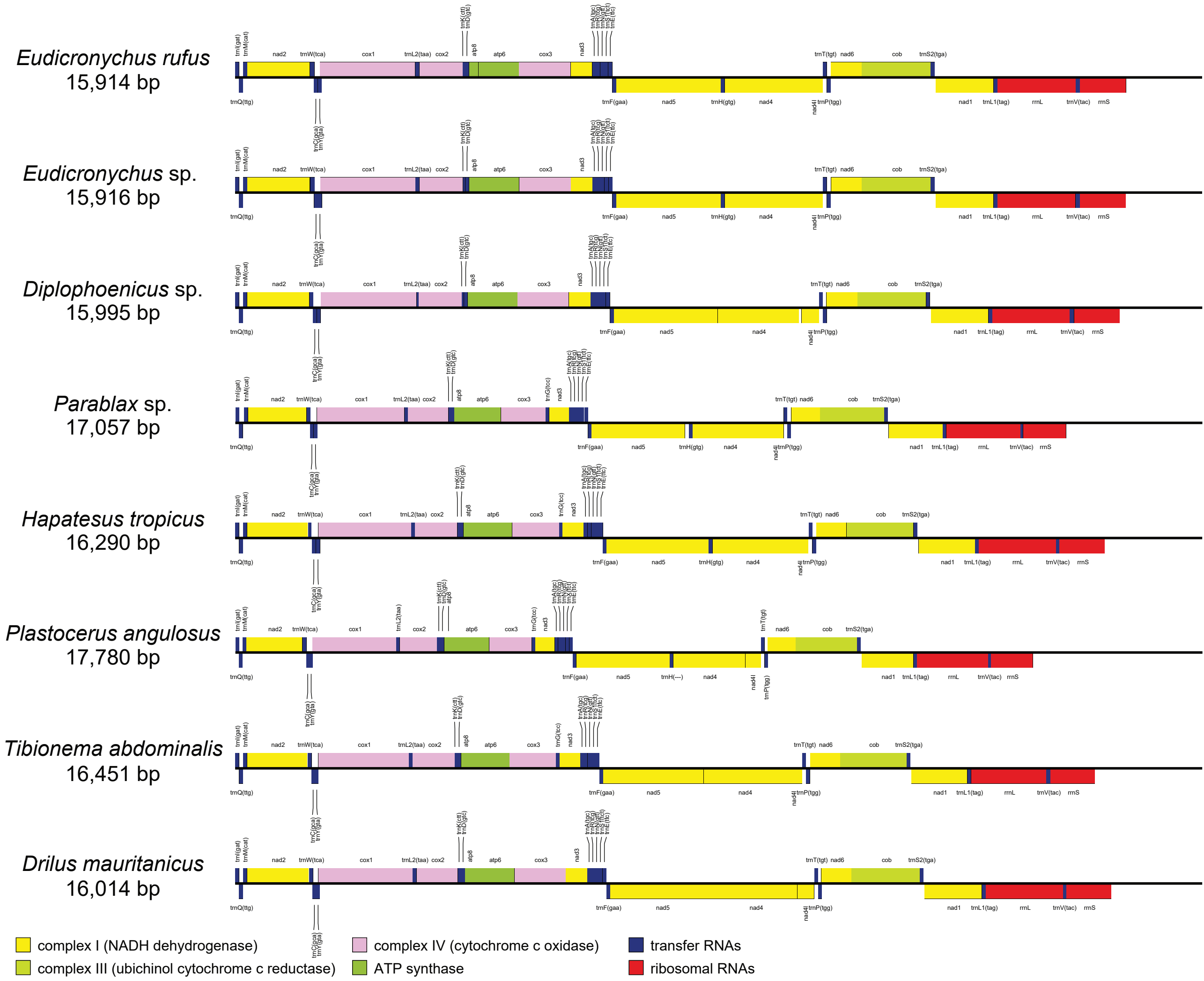

Figure S2. Gene order in newly sequenced linear mitogenomes.

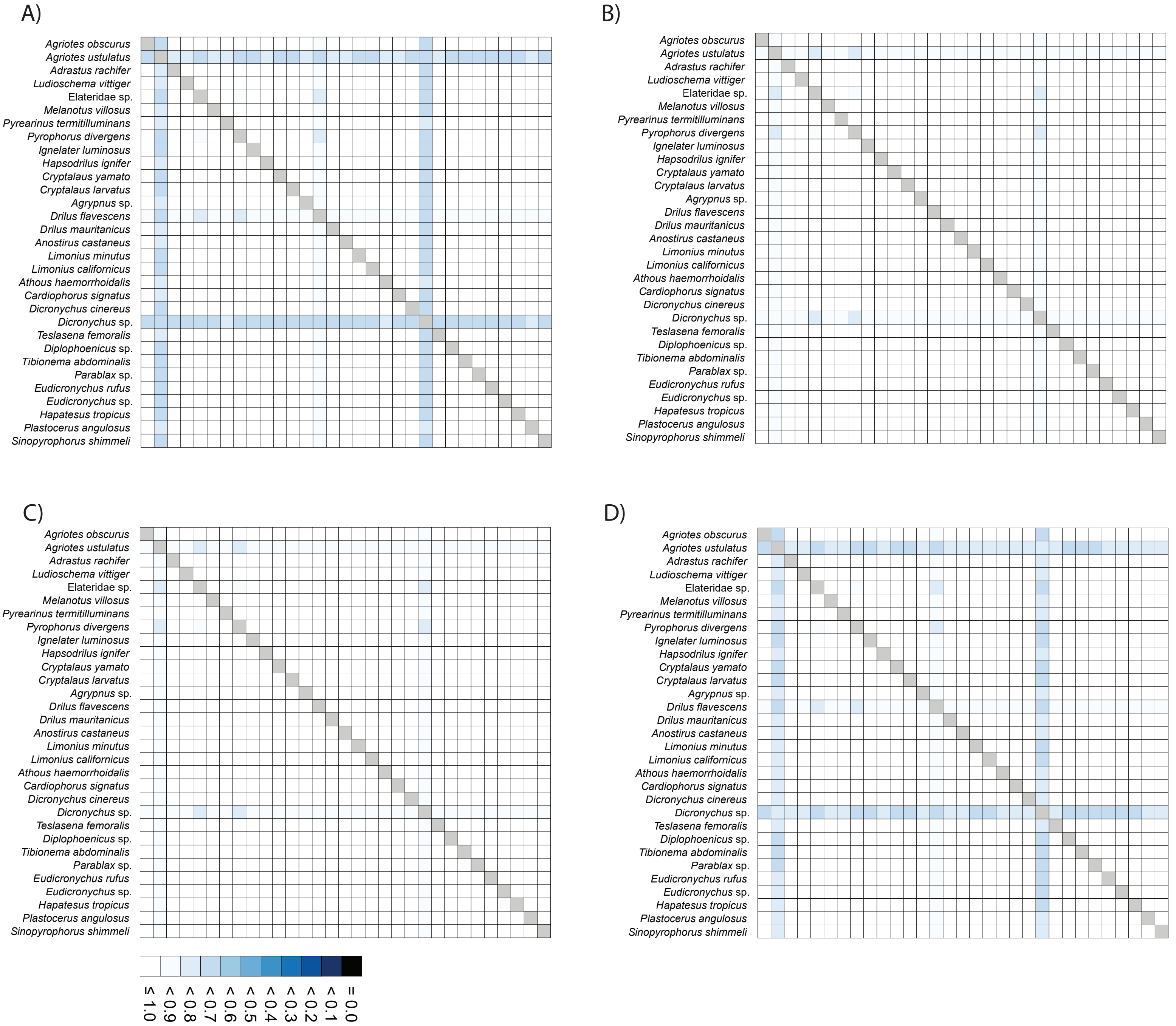

Figure S3. AliStat heat maps of pairwise completeness scores (Ca) in all produced datasets: (A) NUC123: 15 mitochondrial genes (13 PCGs and 2 rRNA), Ca: 0.95; (B) PCN12: 13 mitochondrial PCGs, Ca: 0.97; (C) AA: amino acids of 13 mitochondrial PCGs, Ca: 0.97; (D) MTallAS: dataset A analysed using AliScore, Ca: 0.95.

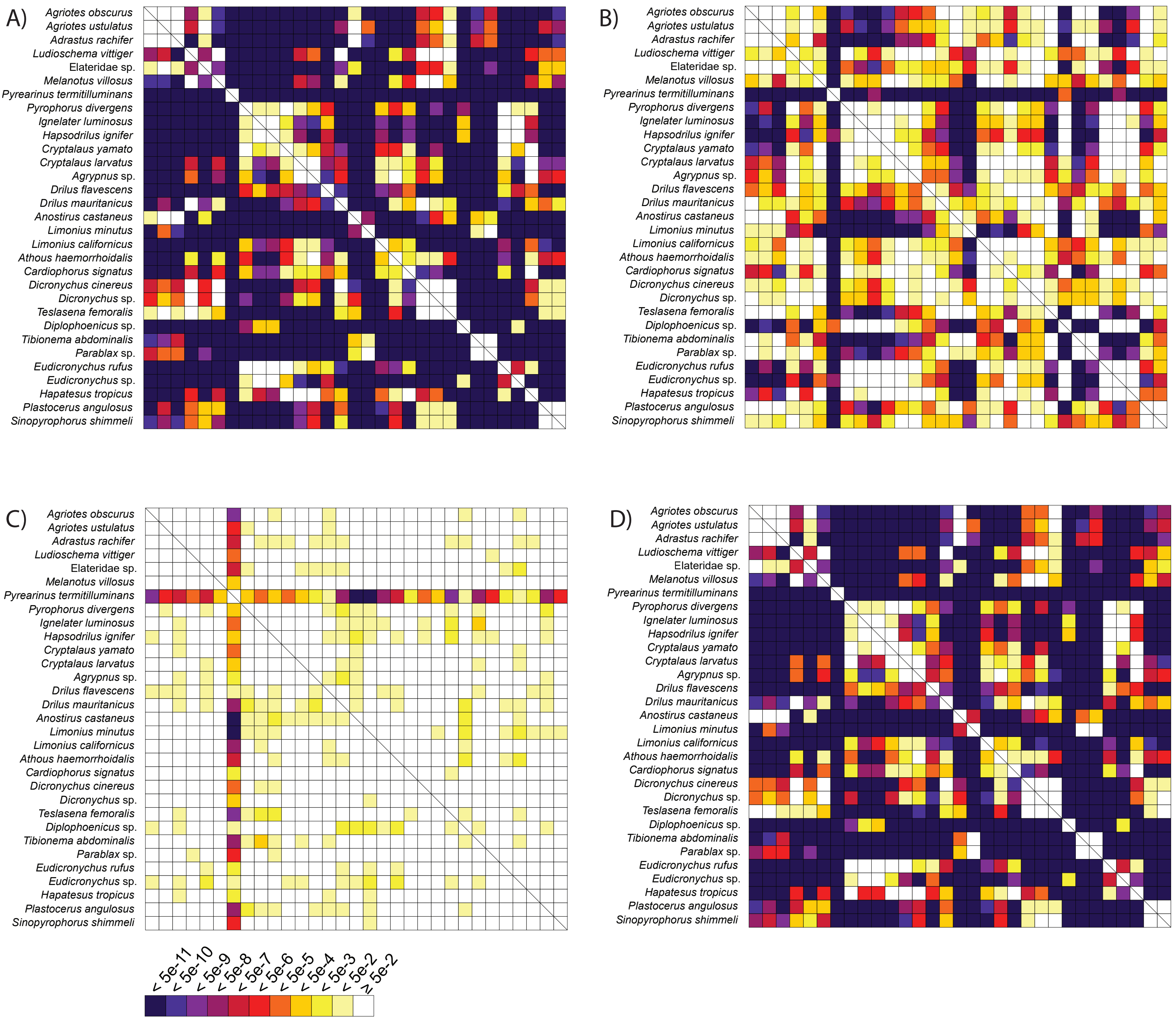

Figure S4. Heat maps calculated with SymTest showing p-values for the pairwise Bowker's tests in in all produced datasets: (A) NUC123: 15 mitochondrial genes (13 PCGs an 2 rRNA), (B) PCN12: 13 mitochondrial PCGs, (C) AA: amino acids of 13 mitochondrial PCGs, (D) MTallAS: dataset A analysed using AliScore.

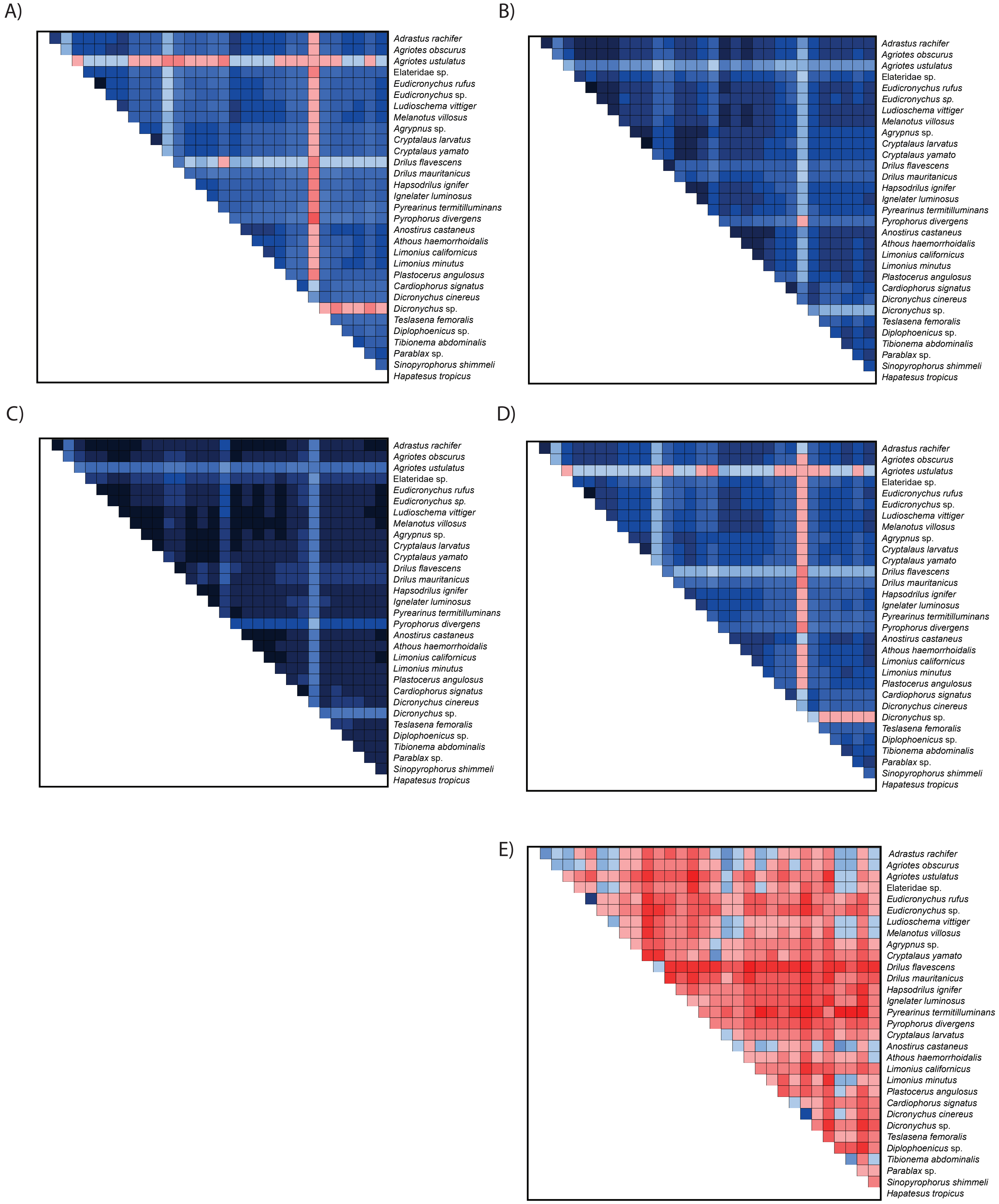

Figure S5. Rectangular heat maps of heterogeneous sequence divergence within sequences in analyzed datasets: (A) NUC123: 15 mitochondrial genes, (B) PCN12: 13 mitochondrial PCGs, (C) AA: amino acids of 13 mitochondrial PCGs, (D) MTallAS: dataset A analysed using AliScore, (E) 13 PCGs third codon position. The mean similarity score between sequences is represented by a coloured square, based on AliGROOVE scores ranging from −1, indicating great difference in rates from the remainder of the data set, i.e. heterogeneity (red), to +1, indicating rates match all other comparisons (blue).

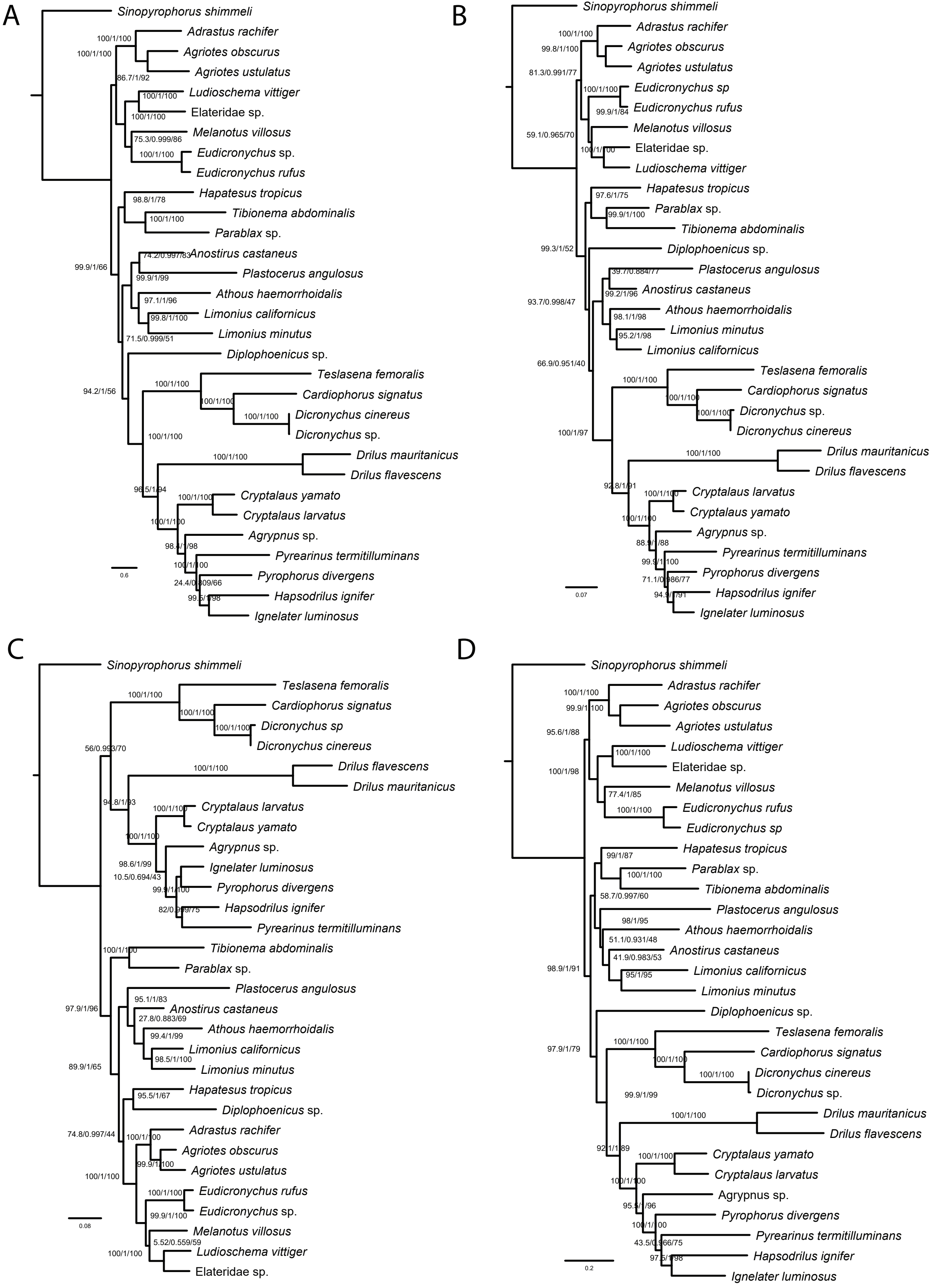

Figure S6. Maximum likelihood trees from IQ-TREE analysis of datasets: **A) NUC123**: 15 mitochondrial genes partitioned by gene and PCGs further partitioned by codon position; **B) PCN12:** 13 mitochondrial PCGs partitioned by gene and by first and second codon positions with third codon position removed; **C) AA:** amino acids of 13 mitochondrial PCGs partitioned by gene; **D) MTallAS:** dataset A analysed using AliScore. The depicted branch support values represent SH-aLRT, abayes test, and ultrafast bootstrap.

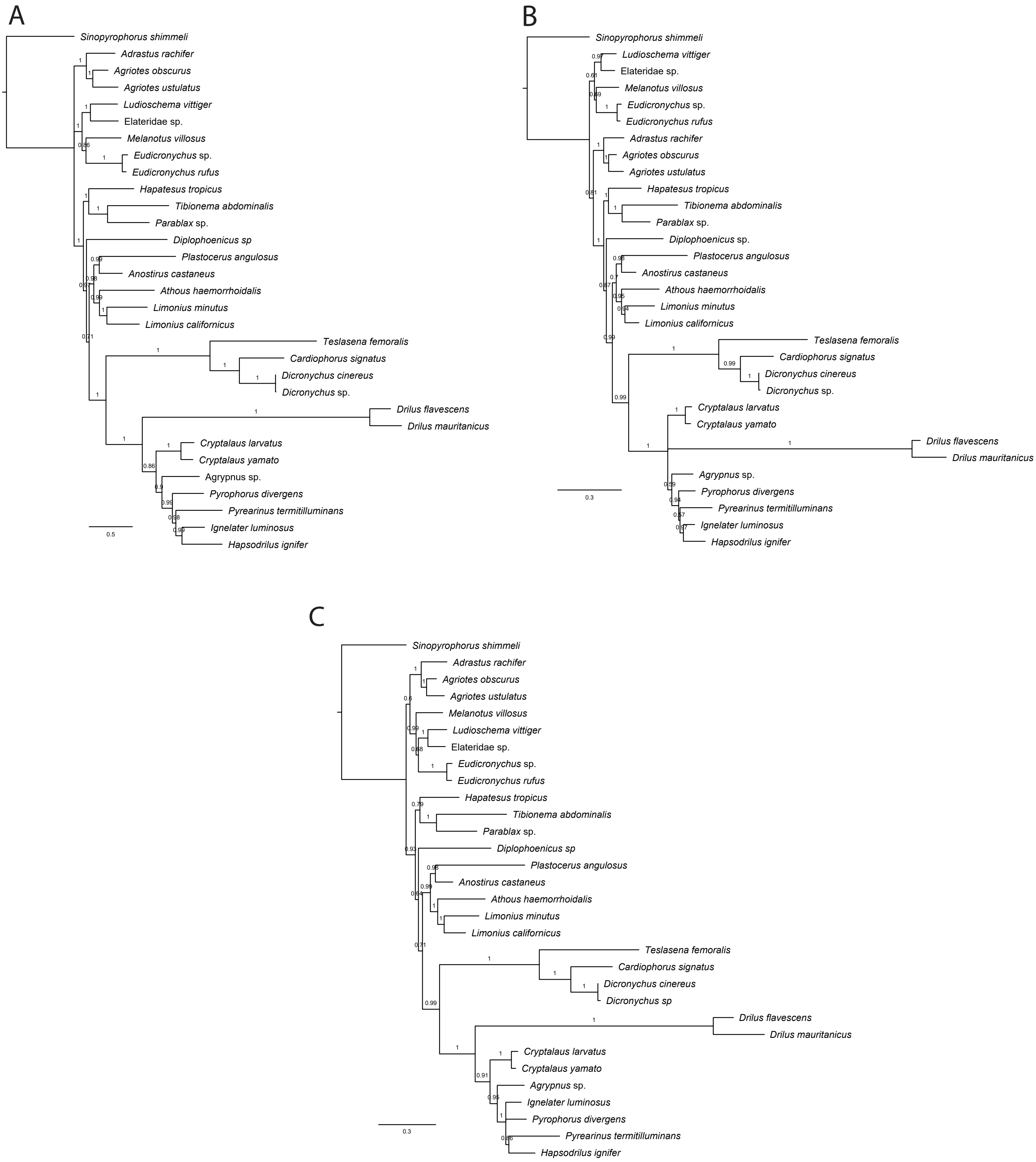

Figure S7. Bayesian trees inferred from unpartitioned datasets: (A) NUC123: 15 mitochondrial genes, (B) PCN12: 13 mitochondrial PCGs and (C) AA: amino acids of 13 mitochondrial PCGs in PhyloBayes under the siteheterogeneous mixture CAT+ GTR model. The values at nodes are Bayesian posterior probabilities.
